# Risk factors for pregnancy-associated heart failure with preserved ejection fraction and adverse pregnancy outcomes: a cross-sectional study

**DOI:** 10.1186/s12884-024-06402-5

**Published:** 2024-03-20

**Authors:** Seon Ui Lee, Jae Young Park, Subeen Hong, Jeong Ha Wie, Jae Eun Shin, Sae Kyung Choi, Woo Jeng Kim, Yeon Hee Kim, Yun Sung Jo, In Yang Park, Kicheol Kil, Hyun Sun Ko

**Affiliations:** 1 Department of Obstetrics and Gynecology, Incheon St. Mary’s Hospital, College of Medicine, The Catholic University of Korea, Seoul, Korea; 2 Department of Obstetrics and Gynecology, College of Medicine, The Catholic University of Korea, Seoul, Korea; 3 Department of Obstetrics and Gynecology, Seoul St. Mary’s Hospital, College of Medicine, The Catholic University of Korea, 222 Banpo-daero, Seocho-Gu Seoul, 137-701 Republic of Korea; 4 Department of Obstetrics and Gynecology, Eunpyeong St. Mary’s Hospital, College of Medicine, The Catholic University of Korea, Seoul, Korea; 5 Department of Obstetrics and Gynecology, Bucheon St. Mary’s Hospital, College of Medicine, The Catholic University of Korea, Seoul, Korea; 6 Department of Obstetrics and Gynecology, Uijeongbu St. Mary’s Hospital, College of Medicine, The Catholic University of Korea, Seoul, Korea; 7 Department of Obstetrics and Gynecology, St. Vincent’s Hospital, College of Medicine, The Catholic University of Korea, Seoul, Korea; 8 Department of Obstetrics and Gynecology, Yeouido St. Mary’s Hospital, College of Medine, The Catholic University of Korea, 10, 63-Ro, Yeongdeungpo-Gu Seoul, Republic of Korea

**Keywords:** Pregnancy, Stroke volume, Heart failure, Pregnancy outcome

## Abstract

**Background:**

Although pregnancy-associated heart failure with preserved ejection fraction (HFpEF) is increasing and contributing to maternal morbidity, little is known about its impact on pregnancy. We examined the risk factors for and adverse pregnancy outcomes of HFpEF in pregnant women.

**Methods:**

We conducted a cross-sectional analysis of pregnancy-related hospitalizations from 2009 to 2020 using the perinatal database of seven multicenters. Cases of HFpEF were identified using the International Classification of Diseases and echocardiography findings. The patients were categorized into the HFpEF and control groups. Risk factors were evaluated using multivariate logistic regression analysis to generate odds ratios (OR) and 95% confidence intervals (CI). Furthermore, adjusted associations between HFpEF and adverse pregnancy outcomes were determined. Risk scores for the stratification of women at a high risk of HFpEF were calculated using a statistical scoring model.

**Results:**

Of the 34,392 women identified, 258 (0.76%) were included in the HFpEF group. In multivariate analysis, HFpEF was significantly associated with old maternal age (OR, 1.04; 95% CI 1.02–1.07), multiple pregnancy (OR, 2.22; 95% CI 1.53–3.23), rheumatic disease (OR, 2.56; 95% CI 1.54–4.26), pregnancy induce hypertension (OR 6.02; 95% CI 3.61–10.05), preeclampsia (OR 24.66; 95% CI 18.61–32.66), eclampsia or superimposed preeclampsia (OR 32.74; 95% CI 21.60–49.64) and transfusion in previous pregnancy (OR 3.89; 95% CI 1.89–8.01). A scoring model to predict HFpEF with those factors achieved an area under the curve of 0.78 at cutoff value of 3. Women with HFpEF also had increased odds ratios of intensive care unit admission during the perinatal period (odds ratio, 5.98; 95% confidence interval, 4.36–8.21) and of postpartum hemorrhage (odds ratio, 5.98; 95% confidence interval, 2.02–3.64).

**Conclusions:**

Pregnancy-associated HFpEF is associated with adverse pregnancy outcomes. A scoring model may contribute to screening HFpEF using echocardiography and preparing adverse pregnancy outcomes.

## Background

Cardiovascular disease (CVD) is one of the most common causes of maternal death and morbidity during pregnancy in developed countries, including the United States [1]. The global prevalence of pregnancy-associated heart failure (HF) has increased over the past several decades [2, 3].

Among the several types of HF, peripartum cardiomyopathy (PPCM) is considered a representative type of HF in pregnant women. It is defined as the new onset of HF with a reduced ejection fraction within the last month of pregnancy or within 5 months after delivery [4]. In South Korea, the incidence of PPCM was reported to be 1 in 1741 deliveries; furthermore, old maternal age, primiparity, and multiple pregnancies were reported to be the risk factors for PPCM in the country [5]. However, because mothers with HF who do not meet the PPCM criteria are generally not evaluated properly, studies worldwide are underway to reevaluate pregnancy-related HF [6–9]. In addition to pregnancy, HF has recently been classified according to the left ventricular ejection fraction. Patients with HF with preserved ejection fraction (HFpEF) and a normal cardiac output account for more than 40% ejection fraction [10]. The clinical diagnosis of HFpEF is based on the following: 1) signs and symptoms of HF, 2) normal range of ejection fraction according to various criteria (i.e., from as low as 40% to as high as 55%), and 3) abnormal left ventricular diastolic function. HFpEF tends to increase with age. Moreover, it is more common in women than in men. It is also a known risk factor for hypertension, obesity, and diabetes [10, 11]. Recent studies have revealed that HFpEF is associated with hospitalization and adverse pregnancy outcomes in pregnant women [6, 12–14]. However, the incidence and risk factors for HFpEF in Asian women have not been thoroughly investigated.

Therefore, the objective of the present study was to evaluate the risk factors and adverse pregnancy outcomes associated with HFpEF in pregnant Asian women.

## Methods

This retrospective cohort study included women who delivered between January 2009 and December 2020 at seven hospitals under the College of Medicine of the Catholic University of Korea. As part of routine obstetric care, obstetricians collect clinical data from electronic medical records (EMR). For this study, data on maternal demographic characteristics and delivery outcomes were collected from the institution’s database via the EMR. Two obstetricians (J.Y.P. and H.S.K.) confirmed the accuracy of data by a chart review.

The exclusion criteria were maternal age < 18 years and pre-existing cardiovascular disease (congenital heart disease, valvular disease, arrhythmia, pulmonary hypertension, cardiomyopathy, and PPCM).

**Fig. 1:**
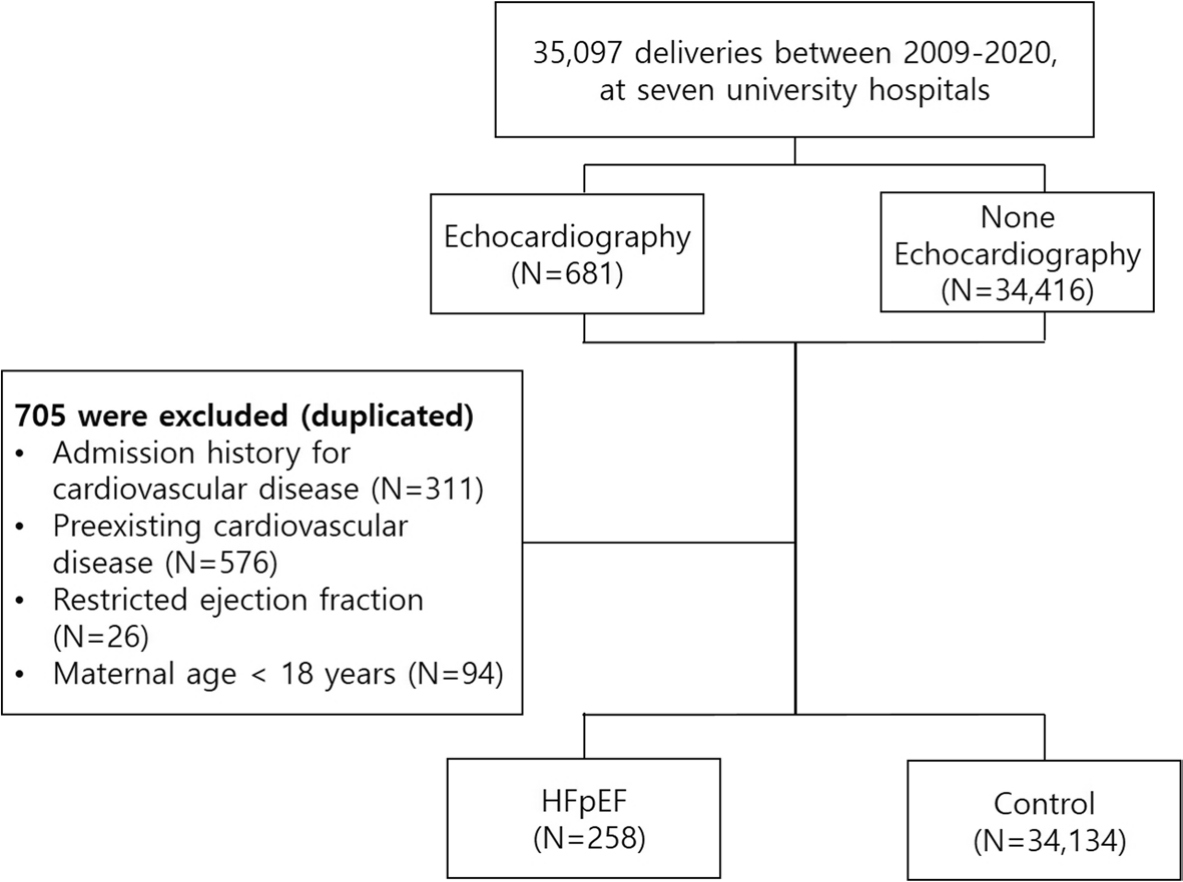
Participant flow chart of the total population

The definition of the HFpEF group in this study was based on the ESC guideline [15]. Women who satisfied the diagnostic criteria for HFpEF within the last month of pregnancy or within 5 months after delivery were included in the HFpEF group; the remaining were included in the control group (Fig. 1).

Data on the following basic maternal characteristics were analyzed: maternal age; body mass index (BMI) before pregnancy and at delivery; parity; fetal number; method of pregnancy; history of smoking; drinking status; preexisting disease including chronic hypertension, pulmonary or arterial embolism, chronic kidney disease (CKD), and rheumatic disease; and history of previous pregnancy including pregnancy-associated hypertension (PAH) or transfusion. Data on the following pregnancy-associated complications were analyzed: PAH, gestational diabetes mellitus (GDM), preterm labor, cesarean section, postpartum bleeding, intensive care unit (ICU) admission, and maternal death. Furthermore, the development of postpartum hypertension or CKD within 6 months of delivery was analyzed. PAH included pregnancy-induced hypertension (PIH), preeclampsia, eclampsia and superimposed preeclampsia. Preterm birth and early preterm birth were defined as delivery at < 37 weeks and < 34 weeks of gestation, respectively. Postpartum bleeding was defined based on ICD code O72 and medical records. In addition, requirement of transfusion, uterine artery embolization, or intrauterine balloon insertion was included in postpartum bleeding.

The Institutional Review Board of the Catholic University of Korea approved this study (XC20WIDI0103). Because this was a retrospective cohort study and because all data were anonymized, the need for informed consent was waived.

### Statistical analysis

Bivariate associations of HFpEF and each predictor variable were evaluated using the chi-square and Fisher’s exact tests for categorical variables and the t-test for continuous variables. Variables with significant differences in the univariate analysis were included in multivariable stepwise logistic regression models. A scoring model for estimating the risk of HFpEF was developed using factors exhibiting independent associations in the multivariate analysis referred to previous risk scoring study [16]. The concordance statistic was computed to assess the model’s ability to discriminate between patients with and without HFpEF. Associations among HFpEF, ICU admission, and postpartum bleeding during the peripartum period were analyzed using univariate and multivariate analyses. All analyses were performed using the Statistical Analysis Software (version 9.4; SAS Institute, Inc., Cary, NC, USA). Statistical significance was indicated by two-sided *P*-values of < 0.05.

## Results

### Baseline characteristics and obstetric outcomes according to HFpEF

Among the 34,392 women included in the study, 258 (0.76%) and 34,134 (99.24%) were categorized into the HFpEF and control groups, respectively (Table 1). The mean maternal age and BMI (both before and at delivery) were significantly higher in the HFpEF group than in the control group (*P* < 0.001). In particular, the proportion of women with a BMI of ≥ 30 kg/m^2^ was higher in the HFpEF group than in the control group (before pregnancy: 10.63% vs. 3.85% [*P* < 0.001]; at delivery: 29.96% vs. 15.85% [*P* < 0.001]). In addition, the mean systolic and diastolic blood pressures at the first visit were higher in the HFpEF group than in the control group (*P* < 0.001). No significant differences between the two groups were noted in terms of the proportion of smokers, drinkers, and women with a history of GDM in previous pregnancies. However, compared with the control group, HFpEF group has significantly higher rates of nulliparity, multiple pregnancies, in vitro fertilization (nullilparity, *P* = 0.003; multiple pregnancies, *P* < 0.001, In vitro fertilization, *P* = 0.004). HFpEF group also showed significantly higher rates of PAH in previous pregnancies, transfusion in previous pregnancies, preexisting disease including chronic hypertension, pulmonary or arterial embolism, CKD, diabetes mellitus, and rheumatic disease (systemic lupus erythematosus, systemic sclerosis, rheumatic arthritis, antiphospholipid syndrome, Sjogren’s syndrome, and Behcet’s disease). (dibetes mellitus, *P* = 0.008; the others, *P* < 0.001). The incidence of adverse pregnancy outcomes of cesarean section, postpartum bleeding, ICU admission, preterm labor, maternal death, PIH, preeclampsia and eclampsia or superimposed preeclampsia was also significantly higher in the HFpEF group than in the control group (maternal death, *P* = 0.002; the others, *P* < 0.001). however, the incidence of GDM did not differ significantly between the two groups (Table 2). Postpartum hypertension and CKD were significantly more prevalent in the HFpEF group than in the control group (*P* < 0.001).

**Table 1:** Baseline characteristics stratified by HFpEF

	HFpEF^a^ (*n* = 258)	Control (*n* = 34,134)	*P*-value
Age, mean (SD)	33.97 (4.57)	32.77 (4.46)	< 0.001
BMI^a^ (kg/m^2^) before pregnancy			
mean (SD)	23.33 (5.04)	21.78 (3.71)	< 0.001
< 25, n (%)	182 (71.65)	28,818 (84.78)	< 0.001
≥ 25, < 30, n (%)	45 (17.72)	3866 (11.37)	
≥ 30, n (%)	27 (10.63)	1308 (3.85)	
BMI^a^ (kg/m^2^) at delivery			
mean (SD)	28.15 (5.43)	26.50 (3.95)	< 0.001
< 25, n (%)	79 (30.74)	13,415 (39.41)	< 0.001
25–30, n (%)	101 (39.30)	15,227 (44.74)	
≥ 30, n (%)	77 (29.96)	5395 (15.85)	
Nulliparity, n (%)	160 (62.02)	18,036 (52.84)	0.003
Multiple pregnancies, n (%)	35 (13.57)	1914 (5.61)	< 0.001
Smoking, n (%)	35 (13.57)	4421 (12.95)	0.770
Drinking, n (%)	31 (12.02)	4148 (12.15)	0.947
In vitro fertilization, n (%)	18 (6.98)	1494 (4.38)	0.042
Chronic hypertension, n (%)	52 (10.08)	2582 (3.78)	< 0.001
History of pulmonary or arterial embolism, n (%)	5 (1.94)	115 (0.34)	0.002
Chronic kidney disease, n (%)	12 (2.33)	160 (0.23)	< 0.001
Transfusion in a previous pregnancy, n (%)	9 (3.49)	287 (0.84)	< 0.001
PAH^a^ in a previous pregnancy, n (%)	20 (7.75)	643 (1.88)	< 0.001
GDM in a previous pregnancy, n (%)	4 (1.55)	730 (2.14)	0.515
Type 1 and Type 2 DM, n (%)	24 (4.65)	1862 (2.73)	0.008
sBP in the first visit, mean (SD)	140.58 (28.97)	118.00 (15.12)	< 0.001
dBP in the first visit, mean (SD)	88.36 (19.42)	74.70 (17.41)	< 0.001
Rheumatic disease^b^	18 (6.98%)	943 (2.76%)	< 0.001

*Note*: Values are expressed as mean (SD) or n (%)

^a^*Abbreviations*: *HFpEF* Heart failure with preserved ejection fraction, *BMI* Body mass index, *PAH* Pregnancy-associated hypertension, *GDM* Gestational diabetes mellitus, *DM* Diabetes mellitus, *sBP* systolic blood pressure, *dBP* Diastolic blood pressure, *SLE* Systemic lupus erythematosus, *APS* Antiphospholipid syndrome, *SD* Standard deviation

^b^Rheumatic disease: corresponding to one among systemic lupus erythematosus, systemic sclerosis, rheumatic arthritis, APS, Sjogren’s syndrome, and Behcet’s disease

**Table 2:** Obstetric outcomes in the HFpEF and control groups

	HFpEF^a^ (*n* = 258)	Control (*n* = 34,134)	*P*-value
PIH^a^, n (%)	18 (6.98)	976 (2.86)	< 0.001
Preeclampsia, n (%)	117 (45.35)	1565 (4.58)	< 0.001
Eclampsia or Superimposed preeclmapsia, n (%)	33 (12.79)	342 (1.00)	< 0.001
GDM^a^, n (%)	21 (8.14)	2669 (7.82)	0.849
Cesarean section, n (%)	203 (78.68)	16,352 (47.91)	< 0.001
Emergency cesarean section, n (%)	27 (10.47)	1600 (4.69)	< 0.001
Postpartum bleeding, n (%)	69 (26.74)	3185 (9.33)	< 0.001
ICU^a^ admission, n (%)	77 (29.84)	750 (2.20)	< 0.001
Duration (SD)	3.22 (4.31)	1.63 (2.74)	< 0.001
Preterm labor before 34 weeks, n (%)	114 (44.19)	3711 (10.87)	< 0.001
Preterm labor before 37 weeks, n (%)	181 (70.16)	8685 (25.44)	< 0.001
Maternal death, n (%)	3 (1.16)	27 (0.08)	0.002
Placental ischemic disease, n (%)	167 (64.73)	5104 (14.95)	< 0.001
Postpartum hypertension, n (%)	82 (35.34)	908 (2.76)	< 0.001
Postpartum chronic kidney disease, n (%)	12 (5.63)	260 (0.85)	< 0.001

*Note*: Values are expressed as mean (SD) or n (%)

^a^*Abbreviations*: *HFpEF* heart failure with preserved ejection fraction, *PIH* pregnancy-induced hypertension, *GDM* gestational diabetes mellitus, *ICU* intensive care unit, *SD* standard deviation

### Risk factors associated with HFpEF in pregnant women

In the univariate analysis, the following factors exhibited significantly increased odds ratios (ORs) with HFpEF: age (OR, 1.07; 95% CI, 1.04**–**1.10), nulliparity (OR, 1.46; 95% CI, 1.13**–**1.88), multiple pregnancies (OR, 2.64; 95% CI, 1.85**–**3.78), pre-pregnancy BMI of ≥ 25 kg/m^2^ (OR, 2.24; 95% CI, 1.69**–**2.97), BMI of ≥ 28 kg/m^2^ at delivery (OR, 1.98; 95% CI, 1.55**–**2.54), in vitro fertilization (OR, 1.64; 95% CI, 1.01**–**2.65), PAH in a previous pregnancy (OR, 4.38; 95% CI, 2.76**–**6.95), chronic hypertension (OR, 2.85; 95% CI, 1.89**–**4.29), history of pulmonary or arterial embolism (OR, 5.85; 95% CI, 2.37**–**14.43), rheumatic disease (OR, 2.64; 95% CI, 1.63**–**4.28), transfusion history in previous pregnancy (OR, 4.26; 95% CI, 2.17**–**8.37), PIH (OR, 6.04; 95% CI, 3.85**–**10.67), preeclampsia (OR, 25.96; 95% CI, 19.63–34.33), eclampsia or superimposed preeclampsia (OR, 33.51; 95% CI 22.17–50.63) and CKD (OR, 10.14; 95% CI, 4.38**–**23.45). However, the multivariate stepwise logistic regression analysis revealed that the following factors were significantly associated with HFpEF (Table 3): PIH (OR, 6.02; 95% CI, 3.61–10.05), preeclampsia (OR, 24.66; 95% CI 18.61–32.66), eclampsia or superimposed preeclampsia (OR 32.74; 95% CI 21.60–49.64), maternal age (OR, 1.04; 95% CI, 1.02–1.07), multiple pregnancies (OR, 2.22; 95% CI, 1.53–3.23), rheumatic disease (OR, 2.56; 95% CI, 1.54–4.26) and transfusion history in previous pregnancy (OR, 3.89; 95% CI, 1.89–8.01).

**Table 3:** Odds ratios for risk factors for HFpEF^a^ from univariate and multivariate stepwise logistic regression analyses

	Univariate analysis	*P*-value	Multivariate analysis	*P*-value
	OR^a^ (95% CI^a^)		OR (95% CI)	
Age	1.07 (1.04, 1.10)	< 0.001	1.04 (1.02, 1.07)	0.003
Nulliparity	1.46 (1.13, 1.88)	0.004		
Multiple pregnancies	2.64 (1.85, 3.78)	< 0.001	2.22 (1.53, 3.23)	< 0.001
BMI^a^ ≥ 25 kg/m^2^ before pregnancy	2.24 (1.69, 2.97)	< 0.001		
BMI ≥ 28 kg/m^2^ at delivery	1.98 (1.55, 2.54)	< 0.001		
Smoking	1.06 (0.74, 1.51)	0.770		
Drinking	0.99 (0.68, 1.44)	0.947		
In vitro fertilization	1.64 (1.01, 2.65)	0.045		
PAH^a^ in previous pregnancy	4.38 (2.76, 6.95)	< 0.001		
Chronic hypertension	2.85 (1.89, 4.29)	< 0.001		
History of pulmonary or arterial embolism	5.85 (2.37, 14.43)	< 0.001		
Chronic kidney disease	10.14 (4.38, 23.45)	< 0.001		
Rheumatic disease^b^	2.64 (1.63, 4.28)	< 0.001	2.56 (1.54, 4.26)	< 0.001
PIH^a^	6.40 (3.85, 10.67)	< 0.001	6.02 (3.61, 10.05)	< 0.001
Preeclampsia	25.96 (19.63, 34.33)	< 0.001	24.66 (18.61, 32.66)	< 0.001
Eclampsia or Superimposed preeclampsia	33.51 (22.17, 50.63)	< 0.001	32.74 (21.60, 49.64)	< 0.001
GDM^a^	1.05 (0.67, 1.64)	0.846		
Type 1 and Type 2 DM^a^	1.74 (0.97, 3.12)	0.063		
Transfusion in previous pregnancy	4.26 (2.17, 8.37)	< 0.001	3.89 (1.89, 8.01)	< 0.001

^a^*Abbreviations*: *HFpEF* heart failure with preserved ejection fraction, *OR* odds ratio, *CI* confidence interval, *BMI* body mass index, *PAH* pregnancy-associated hypertension, *PIH* pregnancy-induced hypertension, *GDM* gestational diabetes mellitus, *DM* diabetes mellitus

^b^Rheumatic disease: corresponding to one among systemic lupus erythematosus, systemic sclerosis, rheumatic arthritis, antiphospholipid syndrome, Sjogren’s syndrome, and Behcet’s disease

### Stratified risk score for estimating the risk of HFpEF in pregnant women

**Fig. 2:**
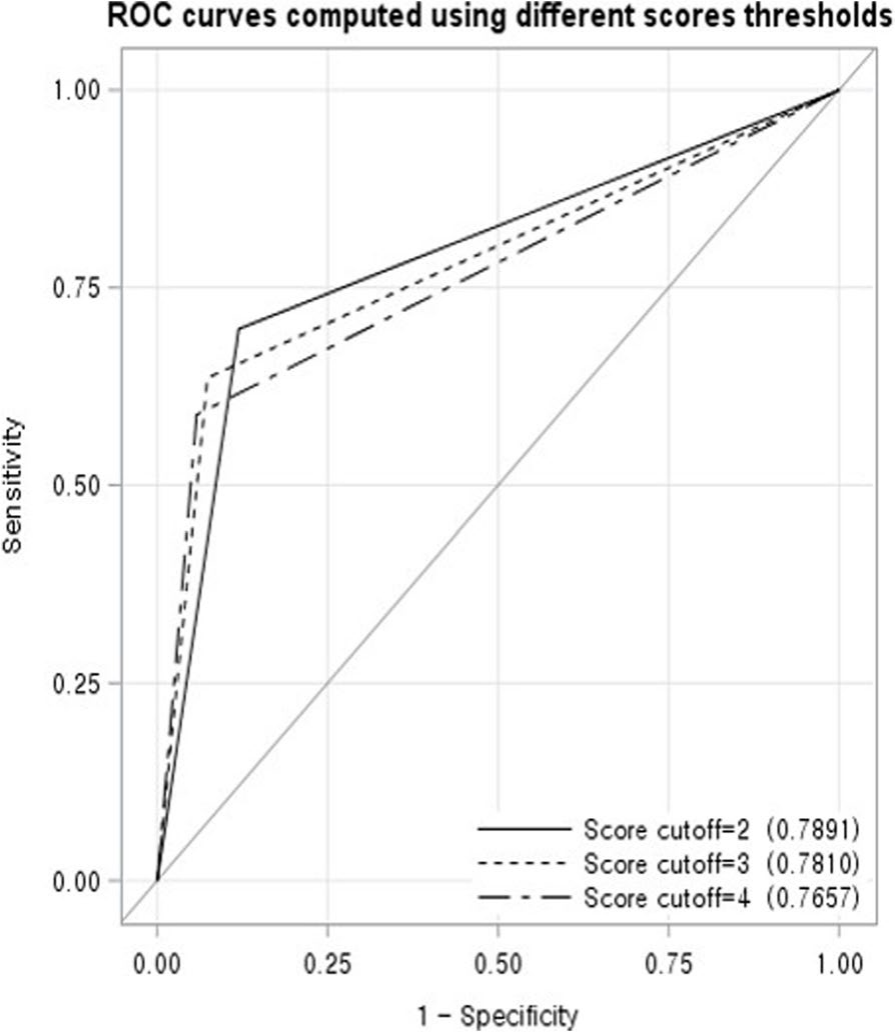
Receiver operating characteristic curves for the scoring model for calculating the risk of HFpEF^a^. Abbreviation: HFpEF, heart failure with preserved ejection fraction

Using the independent risk factors identified in the multivariate analysis, we developed a statistical scoring model to estimate the risk of HFpEF in pregnant women (Table 4). Receiver operating characteristic analysis revealed that for this scoring model, a score cutoff value of 2 demonstrated an area under the curve of 0.79 (sensitivity, 0.70; specificity, 0.88 [*P* < 0.001]) and a score cutoff value of 3 demonstrated an area under the curve of 0.78 (sensitivity, 0.64; specificity, 0.93 [*P* < 0.001]; Fig. 2, Table 5).

**Table 4:** Risk scoring model for HFpEF^a^ in pregnant women

Risk factor		Points
Age		
< 35 years		0
≥ 35 years		1
Multiple pregnancies		
Single		0
Twin, Triplet		1
PAH		
No		0
Yes	PIH^a^	2
	Preeclampsia	4
	Eclampsia or Superimposed preeclampsia	4
Rheumatic disease^b^		
Yes		1
No		0
Transfusion in a previous pregnancy		
Yes		2
No		0
Total points	Estimated risk (%)	
0	0.18%	
1	0.44%	
2	1.03%	
3	2.43%	
4	5.58%	
5	12.32%	
6	25.04%	
7	44.26%	
8	65.37%	
9	82.78%	
10	91.43%	

^a^*Abbreviation*: *HFpEF* heart failure with preserved ejection fraction, *PIH* pregnancy-induced hypertension

^b^Rheumatic disease: corresponding to one among systemic lupus erythematosus, systemic sclerosis, rheumatic arthritis, antiphospholipid syndrome, Sjogren's syndrome, and Behcet's disease

**Table 5:** Cut off scores for calculating the risk of HFpEF^a^

Cutoff score	AUC^a^	Sensitivity	Specificity	PPV^a^	NPV^a^	Accuracy	*P*-value
2	0.789	0.698	0.881	0.042	0.997	0.880	< 0.001
3	0.781	0.636	0.926	0.061	0.997	0.924	< 0.001
4	0.766	0.589	0.942	0.072	0.997	0.940	< 0.001

^a^Abbreviations: *HFpEF* heart failure with preserved ejection fraction, *AUC* area under the receiver operating characteristic curve, *PPV* positive predictive value, *NPV* negative predictive value

### Associations among HFpEF, ICU admission, and postpartum bleeding during the peripartum period

The multivariate stepwise logistic regression analysis (Table 6) revealed that women with HFpEF had a significantly increased OR for ICU admission during the perinatal period (OR, 5.98; 95% CI, 4.36–8.21; *P* < 0.001) and for postpartum hemorrhage (OR, 5.98; 95% CI, 2.02–3.64; *P* < 0.001).

**Table 6:** Multivariate analysis of associations among HFpEF^a^, ICU^a^ admission, and postpartum bleeding during the peripartum period

	Univariate analysis		Multivariate analysis	
	OR^a^ (95% CI)	*P*-value	aOR^a^ (95% CI^a^)*	*P*-value
ICU admission during the peripartum period				
No HFpEF	1		1	
HFpEF	18.94 (14.36, 24.96)	< 0.001	5.98 (4.36, 8.21)	< 0.001
Postpartum bleeding				
No HFpEF	1		1	
HFpEF	3.55 (2.69, 4.69)	< 0.001	5.98 (2.02, 3.64)	< 0.001

^a^*Abbreviations*: *HFpEF* heart failure with preserved ejection fraction, *ICU* intensive care unit, *OR* odds ratio, *aOR* adjusted odds ratio, *CI* confidence interval

^*^ adjusted for maternal age, fetal number, body mass index before pregnancy, body mass index at delivery, in vitro fertilization, drinking history, history of embolism, hypertension, chronic kidney disease, rheumatic disease, history of pregnancy-associated hypertension and postpartum bleeding in the previous pregnancy, pregnancy-associated hypertension, and gestational diabetes mellitus

## Discussion

This study showed that maternal age, multiple pregnancies, rheumatic disease, transfusion in a previous pregnancy and pregnancy-associated hypertension are associated with HFpEF development in pregnant women. Therefore, we created a statistical scoring model using these factors to estimate the risk of HFpEF in pregnant women. Our findings further revealed that HFpEF is significantly associated with ICU admission and postpartum hemorrhage.

The standard definition of HFpEF remains controversial. Recently, most clinicians have considered a combination of the clinical symptoms of HF, a normal or preserved ejection fraction, and structural evidence of cardiovascular abnormalities (including left ventricular hypertrophy and increased left atrial size) to establish an HFpEF diagnosis [17].

In a prospective population-based study, more than 50% of the patients with HF were diagnosed with HFpEF [18]. The hospitalization rate for HFpEF has also increased from 38 to 54% over the past 15 years [19].

The prevalence of HFpEF is higher in women. However, little is known about pregnancy states [20]. Previous studies have mainly focused on HF with reduced ejection fraction, particularly PPCM [19, 21].

In non-pregnant women, the risk factors for HFpEF are obesity, hypertension, elevated fasting glucose levels, metabolic syndrome, and atrial fibrillation. Therefore, correcting modifiable factors, such as through weight loss and treatment for metabolic syndrome, could decrease the incidence of HFpEF in this population [20].

In 2021, a retrospective, cross-sectional analysis of pregnancy-related inpatient hospitalizations was performed using the National Inpatient Sample in the United States. The researchers involved in that investigation described the following as risk factors for pregnancy-associated HFpEF: hypertension (chronic hypertension and hypertensive disorders of pregnancy), anemia, obesity, diabetes, renal disease, and coronary atherosclerosis. They emphasized that women with HFpEF had a 2.61–6.47 times greater risk of adverse pregnancy outcomes. Compared to women without HFpEF, they were more likely to experience hypertensive disorders, stillbirth, fetal growth restriction, preterm labor, and cesarean delivery. However, the researchers concluded that the diagnosis and prediction of pregnancy-associated HFpEF are challenging because of multiple phenotypes and physiological changes during pregnancy [13].

Other than the aforementioned study, only case reports and series on pregnancy-associated HFpEF have been published [9, 14, 22–24]. These reports have described the adverse outcomes in women with HFpEF or PPCM; they have further indicated that echocardiographic measurement of diastolic dysfunction, such as through atrial strain imaging, could help in the diagnosis of HFpEF.

Hypertension is the most common comorbidity of HFpEF in non-pregnant women [17]. The incidence of CVD (coronary artery disease, HF, aortic stenosis, and mitral regurgitation) increases with prior hypertensive disorders of pregnancy [25]. In a cross-sectional study, a hypertensive disorder of pregnancy was associated with an impaired diastolic phenotype and HFpEF. The increase in HFpEF mirrors the increasing prevalence of hypertension and hypertensive disorders during pregnancy [13]. Similarly, in this study, PAH was included as an independent risk factor for HFpEF and assigned the highest score in the scoring system. Additionally, preeclampsia, and eclampsia or superimposed preeclampsia had greater impacts on the development of HFpEF. Therefore, routine echocardiography may be required in women with PAH.

Standard treatment for HFpEF has not yet been established. Furthermore, treatment options are considerably limited for pregnant women with HFpEF. Using a statistical risk-scoring model for HFpEF, we can predict the condition in advance in pregnant women and prepare for its prevention and treatment; this may help improve the prognosis and pregnancy outcomes.

The limitation of our study is the small number of patients with HFpEF (*n* = 258) in a retrospective cohort. In addition, selection bias cannot be ruled out because maternal echocardiography was not performed in all cases; it was only performed in certain populations with hypertension, dyspnea, and borderline or abnormal findings on chest radiography or electrocardiogram.

However, our study also has several strengths. First, the samples were obtained from seven multicenters in different regions. Second, we excluded women with pre-existing heart disease who had an underlying risk of HFpEF. Finally, we developed a scoring system that can be applied while consulting individual patients. Echocardiographic examination and close monitoring of pregnant women with scores of > 3 in this model may help plan their peripartum management to decrease maternal morbidity.

## Conclusions

The prevalence of HFpEF is increasing; however, little is known about maternal HFpEF. Compared to women without HFpEF, women with HFpEF are more likely to experience poor obstetric and peripartum outcomes. A scoring model may be beneficial for screening and diagnosing HFpEF using echocardiography, enabling its management for decreasing adverse pregnancy outcomes.

## Data Availability

Data is provided within the manuscript or supplementary information files.

## Abbreviations

OR: Odds ratio
CI: Confidence interval
HFpEF: Heart failure with preserved ejection fraction
ICU: Intensive care unit
CVD: Cardiovascular disease
HF: Heart failure
PPCM: Peripartum cardiomyopathy
BMI: Body mass index
CKD: Chronic kidney disease
GDM: Gestational diabetes mellitus
PAH: Pregnancy-associated hypertension
PIH: Pregnancy-induced hypertension
